# Validation of two PROMIS item banks for measuring social participation in the Dutch general population

**DOI:** 10.1007/s11136-018-1995-0

**Published:** 2018-09-10

**Authors:** C. B. Terwee, M. H. P. Crins, M. Boers, H. C. W. de Vet, L. D. Roorda

**Affiliations:** 1 Department of Epidemiology and Biostatistics, Amsterdam Public Health Research Institute, VU University Medical Center, P.O. Box 7057, 1007 MB Amsterdam, The Netherlands; 2 Amsterdam Rehabilitation Research Center | Reade, Amsterdam, The Netherlands; 3 Amsterdam Rheumatology and Immunology Center, VU University Medical Center, Amsterdam, The Netherlands

**Keywords:** Participation, Validation, PROMIS, IRT

## Abstract

**Background:**

The Patient-Reported Outcomes Measurement Information System (PROMIS) item banks ‘Ability to Participate in Social Roles and Activities’ (35 items) and ‘Satisfaction with Social Roles and Activities’ (44 items) were developed to measure (satisfaction with) participation more efficiently and precisely than current instruments, by using Computerized Adaptive Testing (CAT). We validated these item banks in a Dutch general population.

**Methods:**

Participants in an internet panel completed both item banks. Unidimensionality, local dependence, monotonicity, Graded Response Model item fit, Differential Item Functioning (DIF) for age, gender, education, region, ethnicity, and language (Dutch compared to US Social Supplement), and reliability were assessed.

**Results:**

A representative Dutch sample of 1002 people participated. We found for the Ability to Participate and Satisfaction with Participation item banks, respectively, sufficient unidimensionality (CFI: 0.971, 0.960; TLI: 0.970, 0.958; RMSEA: 0.108, 0.108), no local dependence, sufficient monotonicity (H: 0.75, 0.73), good item fit (2 out of 35 items, 1 out of 44 items with S-X^2^*p*-value < 0.001). No DIF was found. We found a reliability of at least 0.90 with simulated CATs in 86% and 94% of the participants with on average 4.7 (range 2–12) and 4.3 (range 3–12) items, respectively.

**Discussion:**

The PROMIS participation item banks showed sufficient psychometric properties in a general Dutch population and can be used as CAT. PROMIS CATs allow reliable and valid measurement of participation in an efficient and user-friendly way with limited administration time.

## Introduction

Participation in social roles and activities is a major determinant of many favorable health and quality of life outcomes, and a key dimension of successful aging [1, 2]. The *International Classification of Functioning, Disability and Health (ICF)* distinguishes participation from activity limitations (or physical function) and defines participation as “an individual’s involvement in life situations in relation to health conditions, body functions and structure, activities, and contextual factors” [3]. Social participation declines as a result of ‘normal’ aging [4] and increasing morbidity. It is important to monitor social participation in populations and individual patients to develop and evaluate interventions that can improve social participation, for example in elderly and patients with chronic diseases [5–7], and to measure participation as an outcome in clinical trials [8, 9].

Many Patient-Reported Outcome Measures (PROMs) are available to measure participation, both generic and disease-specific [8, 10–14], but their use is associated with a number of challenges. The instruments vary in content and operationalization of the concept of participation [10–12] and in their measurement properties [11, 15–17]. Scores of different instruments are incomparable because the scales are ordinal and even if scores are expressed on a scale from 0 to 100, one cannot assume that a score of 40 points on one instrument corresponds to a score of 40 points on another instrument. Furthermore, many instruments are considered to be too long for use in daily clinical practice.

The Patient-Reported Outcomes Measurement Information System (PROMIS) initiative developed two universally applicable item banks to measure participation, defined as performing one’s social roles and activities: the PROMIS item bank ‘Ability to Participate in Social Roles and Activities’ and the PROMIS item bank ‘Satisfaction with Social Roles and Activities’ [18, 19]. The item banks were developed based on modern psychometric methods (Item Response Theory (IRT)) to overcome the above-mentioned challenges.

PROMIS item banks have several advantages compared to traditional PROMs. One of the main advantages is that PROMIS item banks can be administered as Computerized Adaptive Test (CAT). In CAT, after the first item, the selection of subsequent items is determined by the person’s responses to the previous items. With CAT, persons need to complete on average only 3–7 items to get a reliable score, compared to 20–30 items with a traditional questionnaire [20, 21]. This makes PROMIS CATs highly suitable for use in studies, alongside other instruments, as well as in daily clinical practice. Fixed short forms of subsets of 4–8 items are also available for applications where a computer is not available. Short forms of the Ability to Participate item bank are included in PROMIS Profile instruments [22], and in preference measures [23, 24]. Another advantage of PROMIS is that the instruments are applicable across (disease) populations. The interest in universally applicable instruments is rising, given the increasing number of people with multiple chronic diseases [25–28].

Good psychometric properties of the PROMIS participation item banks were found in US and Spanish general populations. Both language versions were considered unidimensional (Comparative Fit Index (CFI) 0.97 for the Ability item bank in English and Spanish and 0.96 and 0.94 for the Satisfaction item bank in English and Spanish, respectively), all items fitted the IRT model and item parameters were considered similar (i.e., no Differential Item Functioning (DIF)) across gender, age, and education [19]. Evidence for internal consistency, test–retest reliability, construct validity, and responsiveness was found for the short forms in patients with rheumatoid arthritis, osteoarthritis, fibromyalgia, systemic lupus erythematosus, systemic sclerosis, idiopathic pulmonary fibrosis, and patients undergoing cervical spine surgery [29–34].

The PROMIS participation item banks were recently translated into Dutch-Flemish [35]. The aim of this study was to validate these two item banks in a general Dutch population.

## Methods

### Participants

We used an existing internet panel of the general Dutch population polled by a certified company (Desan Research Solutions). We deemed a sample of at least 1000 people sufficient for item parameter estimation. The sample should be representative of the Dutch general population (maximum of 2.5% deviation) with respect to distribution of age (18–40; 40–65; > 65), gender, education (low, middle, high), region (north, east, south, west), and ethnicity (native, first, and second generation western immigrant, first and second generation non-western immigrant), based on data from Statistics Netherlands in 2016 [36].

### Measures

The item bank Ability to Participate in Social Roles and Activities assesses the perceived ability to perform one’s usual social roles and activities. All 35 items are worded in terms of perceived limitations, e.g., “I have trouble doing my regular daily work around the house” and scored on a 5-point Likert response scale (never, rarely, sometimes, usually, always). Responses are reverse-coded so that higher scores represent better ability. The item bank Satisfaction with Social Roles and Activities assess satisfaction with performing one’s usual social roles and activities, e.g., “I am satisfied with my ability to participate in family activities.” All 44 items are scored on a 5-point Likert response scale (not at all, a little bit, somewhat, quite a bit, very much) with higher scores representing more satisfaction. There is no time frame in any of the items.

### Procedure

Participants completed all 35 items of the Dutch-Flemish V2.0 PROMIS item bank Ability to Participate in Social Roles and Activities and all 44 items of the Dutch-Flemish V2.0 PROMIS item bank Satisfaction with Social Roles and Activities through an online survey. In addition, participants completed general questions about age, gender, education, region, and ethnicity.

### Statistical analysis

We conducted psychometric analyses in accordance with the PROMIS analysis plan [37]. An IRT model requires that three assumptions are met: unidimensionality, local independence, and monotonicity.

First, we examined unidimensionality by Confirmatory Factor Analyses (CFA) on the polychoric correlation matrix with Weighted Least Squares with Mean and Variance adjustment (WLSMV) estimation, using the R package LAVAAN (version 0.5-23.1097) [38]. For unidimensionality, all items must load on a single factor. The CFI, Tucker Lewis Index (TLI), Root Means Square Error of Approximation (RMSEA) and Standardized Root Mean Residual (SRMR) evaluated model fit. We report scaled fit indices, which are considered more exact than unscaled indices [39, 40]. In addition, we performed an Exploratory Factor Analysis (EFA) on the polychoric correlation matrix with WLSMV estimation procedures using the R package Psych (version 1.7.5) [41]. Following the PROMIS analysis plan and recommendations from Hu and Bentler [42], we considered sufficient evidence for unidimensionality if CFI > 0.95, TLI > 0.95, RMSEA < 0.06, SRMR < 0.08, the first factor in EFA accounted for at least 20% of the variability, and the ratio of the variance explained by the first to the second factor was greater than four [37, 43].

Second, we evaluated local independence. After controlling for the dominant factor, there should be no significant covariance among item responses. We examined the residual correlation matrix resulting from the single factor CFA mentioned above, and considered residual correlations greater than 0.20 indicators of possible local dependence [37].

Third, we assessed monotonicity. The probability of endorsing a higher item response category should increase (or at least not decrease) with increasing levels of the underlying construct [37]. We evaluated monotonicity by fitting a non-parametric IRT model, with Mokken scaling, using the R-package Mokken (version 2.8.4) [44, 45]. This model yields non-parametric IRT response curve estimates, showing the probabilities of endorsing response categories that can be visually inspected to evaluate monotonicity. We evaluated the fit of the model by calculating the scalability coefficient H per item and for the total scale. We considered monotonicity acceptable if the scalability coefficients of the items were at least 0.30, and the scalability coefficient for the total scale was at least 0.50 [45].

To study IRT model fit, we fitted a logistic Graded Response Model (GRM) to the data using the R-package Mirt (version 3.3.2) [46]. A GRM models two kind of item parameters, item slopes and item thresholds. The item slope refers to the discriminative ability of the item, with higher slope values indicating better ability to discriminate between adjoining values on the construct. Item thresholds refer to item difficulty, and locate the items along the measured trait (i.e., the construct of interest) [47]. For items with five response categories, four item thresholds are estimated. To assess the fit of the GRM model, we used a generalization of Orlando and Thissen’s S-X^2^ for polytomous data [48]. This statistic compares observed and expected response frequencies under the estimated IRT model, and quantifies the differences between them. The criterion for good fit of an item is a S-X^2^*p*-value greater than 0.001 [49].

We used DIF analyses to examine measurement invariance, i.e., whether people from different groups (e.g., males vs females) with the same level of the trait (participation) have different probabilities of giving a certain response to an item [50]. We evaluated DIF for age (median split: ≤ 53 years, > 53 years), gender (male, female), education (low, middle, high), region (north, east, south, west), ethnicity (native, first and second generation western immigrant, first and second generation non-western immigrant), and language (English vs Dutch). For this latter aim, we compared our sample to the US PROMIS 1 Social Supplement, obtained from the HealthMeasures Dataverse repository [51], which was used to develop these item banks [18]. We selected only the participants from this Supplement who were recruited from the US general population (Polimetrix sample, *n* = 1008). From this group, we used 429 people with complete data for the Ability to Participate in Social Roles and Activities item bank and 424 people with complete data for the Satisfaction with Social Roles and Activities item bank for the DIF analyses. We evaluated DIF by a series of ordinal logistic regression models, using the R package Lordif (version 0.3-3) [52], which models the probability of giving a certain response to an item as a function of the trait, a (dichotomous or ordinal) group variable, and the interaction between the trait and the group variable. We used a McFadden’s pseudo *R*^2^ change of 2% between the models as a criterion for DIF [52]. Uniform DIF exists when the magnitude of the DIF is consistent across the entire range of the trait. Non-uniform DIF exists when the magnitude or direction of DIF differs across the trait.

Finally, we evaluated reliability. Reliability within IRT is conceptualized as “information.” Information (I) is inversely related to the standard error (SE) of the estimated construct or trait level (called theta, *θ*), as indicated by the formula:

$${\text{SE}}(\theta )=\frac{1}{{\sqrt {I(\theta )} }}.$$

The SE can differ across theta [47, 53]. The theta is estimated based on the GRM model and scaled with a mean of 0 and a SD of 1 and an effective range of − 4 to 4. An SE of 0.316 corresponds to a reliability of 0.90 (SE 0.548 corresponds to a reliability of 0.70). For each person, we calculated four theta scores: one based on all items of the item bank, one based on the standard 8-item short form (version 8a), and two based on different CAT simulations. In the first simulated CAT, we used the standard PROMIS CAT stopping rules. The standard CAT stopped when a SE of 3 on the *T*-score metric was reached (comparable to a reliability slightly higher than 0.90) or a maximum of 12 items was administered (the recommended minimum by PROMIS is 4 items, but this could not be defined in catR so we used no minimum). In the second simulated CAT, we administered a fixed number of 8 items to compare the reliability of the CAT with the short form. In all simulations, the starting item was the item with the highest information value for the average level of participation in the population (*θ* = 0), according to PROMIS practice. We used the R-package catR (version 3.12) for the CAT simulations [54]. We used maximum likelihood (ML) for estimating thetas to prevent biased scores in people with extreme responses [55]. ML, however, is not able to estimate *θ* for response patterns that exclusively comprise extreme responses. Therefore, we set the possible scale boundaries in the CAT simulation to − 4 to 4, whereby people who score 1 or 5 on all CAT items get a theta score of − 4 or 4.

We transformed theta scores into *T*-scores as recommended by PROMIS according to the formula (θ*10) + 50. A *T*-score of 50 represents the average score of the study population, with a standard deviation of 10. We plotted the SE across *T*-scores for the entire item banks, for the standard 8-item short forms (version 8a), and for the two different CAT simulations [54]. We plotted the distribution of *T*-scores in our population to show the reliability of the item bank in relation to the distribution of scores in the population.

## Results

A sample of 1002 people from the panel completed the questionnaire (mean age 51 (SD 17), 52% female) between July and November 2016. All participants had complete data. The demographic characteristics of the participants are summarized and compared to the Dutch general population in 2016 in Table 1. All differences were less than the 2.5% agreed upon.

**Table 1:** Characteristics of the participants

	Dutch general population study sample	Dutch general population 2016^a^	Difference between study sample and population in 2016	US sample^b^
	*N* (%)	%	%	*N* (%)
*Number of patients*	1002	13,562,539		1008
Age (years)				
18–39	316 (31.5)	33.7	–2.2	
40–65	457 (45.6)	43.6	2.0	
> 65	229 (22.9)	22.7	0.2	
*Mean* (*SD*) *in years*	51 (17)			56 (15)
Gender				
Male	477 (47.6)	49.2	–1.6	336 (40.8)
Female	525 (52.4)	50.8	1.6	487 (59.2)
Education^c^				
Low	294 (29.3)	30.2	–0.9	3 (0.3)
Middle	427 (42.6)	40.2	2.4	171 (17.0)
High	281 (28.0)	29.6	–1.6	823 (81.6)
Region^d^				
North	102 (10.2)	10.2	0.0	
East	199 (19.9)	20.8	–0.9	
South	201 (20.1)	21.6	–1.5	
West	497 (49.6)	47.4	2.2	
Ethnicity				
Native	774 (77.2)	78.6	–1.4	White: 74.4%
First and second generation western immigrant	127 (12.6)	10.3	2.4	
First and second generation non-western immigrant	101 (10.1)	11.2	–1.1	

^a^Based on data from statistics Netherlands (http://www.cbs.nl)

^b^Of the total sample of 1008, we used 429 people with complete data for the Ability to Participate in Social Roles and Activities item bank and 424 people with complete data for the Satisfaction with Social Roles and Activities item bank. Number of missing values: age: 6, gender: 185, education: 185, ethnicity: 188

^c^Low = primary school, lower levels of secondary school (in Dutch: VMBO or lower), lower vocational education; middle = higher levels of secondary school (in Dutch: HAVO/VWO), middle vocational education; high = at least first year of bachelor degree

^d^Three missing values in the Dutch sample

### Ability to Participate in Social Roles and Activities

The three assumptions for fitting an IRT model were considered to be met. The scaled CFA fit indices were CFI: 0.97, TLI: 0.97, RMSEA: 0.11, and SRMR: 0.04. In EFA, the eigenvalue of the first factor was 27.3; the eigenvalue of the second factor was 0.92 (ratio 29.7). These results were considered showing enough evidence for unidimensionality. No item pairs were flagged for local dependence. The Mokken scalability coefficients of the items were ≥ 0.30 (range 0.64–0.79) and the scalability coefficient of the full item bank was 0.75, supporting monotonicity.

Four out of 35 items had a poor item fit in the GRM model, with S-X^2^*p*-value of less than 0.001 (RP1 “I have trouble doing my regular daily work around the house,” SRPPER09_CaPS “I have trouble doing everything for work that I want to do (include work at home),” SRPPER17r1 “I feel limited in the amount of time I have for my family,” and SRPPER28r1 “I have to limit my regular activities with friends”). The item slope parameters ranged from 2.4 to 4.8, with mean of 3.9. The item with lowest slope (worst discriminative ability) was SRPPER43r1 (“I have trouble keeping in touch with others”), and the item with the highest slope (best discriminative ability) was SRPPER08_CaPS (“I have trouble doing all of the family activities that are really important to me”). The item threshold parameters ranged from − 2.5 to 0.6. No items were flagged for DIF for age, gender, education, region, ethnicity, or language.

Based on the GRM model with ML estimation, a theta could not be estimated for 77 (7.7%) of the participants because they had extreme scores. The item with the highest information at *θ* = 0 (average of the population) was SRPPER08_CaPS “I have trouble doing all of the family activities that are really important to me.” This item was used as a starting item in the CAT simulations.

**Fig. 1:**
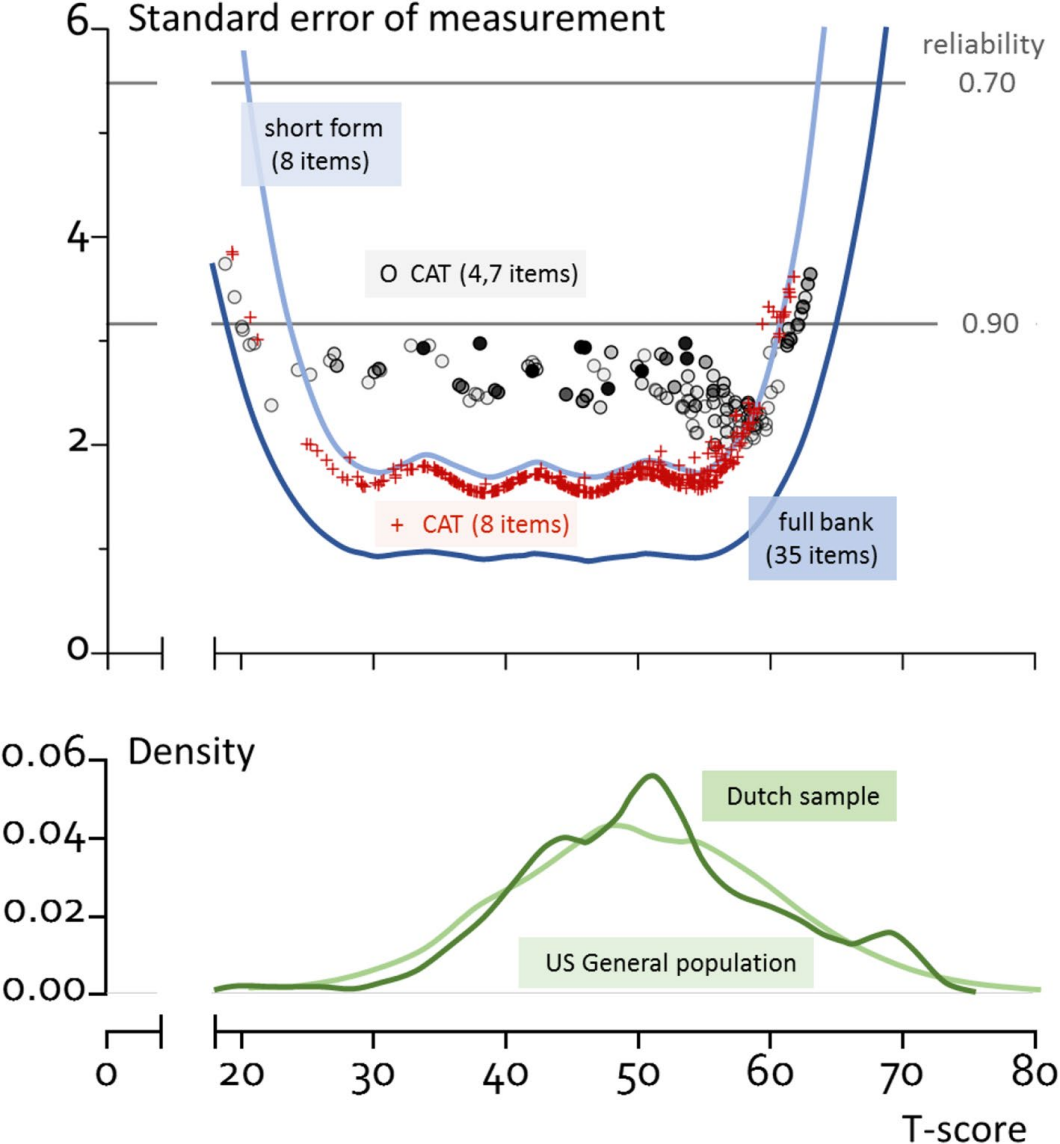
Reliability of the PROMIS V2.0 item bank Ability to Participate in Social Roles and Activities when using different applications (full item bank, short form and simulated CATs (the open circle represent the standard CAT (shading represents many of the same scores) and the plus symbols represent the fixed 8-item CAT)) and distribution of *T*-scores (based on full item bank) in the population. For *n* = 77, theta could not be estimated and CAT theta scores were set to − 4 (*T*-score 10, *n* = 7) or 4 (*T*-score 90, *n* = 70) in both simulated CATs (these persons are not shown in the plot)

Figure 1 shows the standard error across *T*-scores for the full item bank, the short form, and the two simulated CATs. The full item bank (35 items) had a reliability of > 0.90 for 92% of the participants. The short form (8 items) had a reliability of > 0.90 for 85% of the participants. Using the standard CAT with SE = 3 and max 12 items, a reliability of > 0.90 was obtained for 86% of the participants with an average of 4.7 (range 2–12) items. Using a fixed 8-item CAT, a reliability of > 0.90 was obtained for 82% of the participants.

The mean *T*-score of the study sample (based on the full item bank, obtained from https://www.assessmentcenter.net/ac_scoringservice, using US item parameters) was 50.6 (SD 9.5), range of 20.5–69.3.

### Satisfaction with social roles and activities

The three assumptions for fitting an IRT model were considered to be met. The scaled CFA fit indices were CFI: 0.96, TLI: 0.96, RMSEA: 0.11, and SRMR: 0.05. In EFA, the eigenvalue of the first factor was 33.0; the eigenvalue of the second factor was 1.44 (ratio 22.9). These results were considered showing enough evidence for unidimensionality. No item pairs were flagged for local dependence. The Mokken scalability coefficients of the items were ≥ 0.30 (range 0.66–0.77), and the scalability coefficient of the full item bank was 0.73, supporting monotonicity.

Two out of 44 items had a poor item fit in the GRM model, with a S-X^2^*p*-value of less than 0.001 (SRPSAT24r1 “I am satisfied with my ability to work (include work at home),” and SRPSAT51r1 “I am satisfied with my ability to run errands”). The item slope parameters ranged from 2.3 to 4.2, with mean of 3.5. The item with lowest slope (worst discriminative ability) was SRPSAT48r1 “I am satisfied with my ability to do things for fun at home (like reading, listening to music, etc.),” and the item with the highest slope (best discriminative ability) was SRPSAT29_CaPS “I am satisfied with my ability to engage in activities with friends.” The item threshold parameters ranged from − 2.1 to 1.6. None of the items were flagged for DIF for age, gender, education, region, ethnicity, or language.

Based on the GRM model with ML estimation, a theta could not be estimated for 28 (2.8%) of the participants because they had extreme scores. The item with the highest information at *θ* = 0 (average of the population was SRPSAT29_CaPS) “I am satisfied with my ability to engage in activities with friends.” This item was used as a starting item in the CAT simulations.

**Fig. 2:**
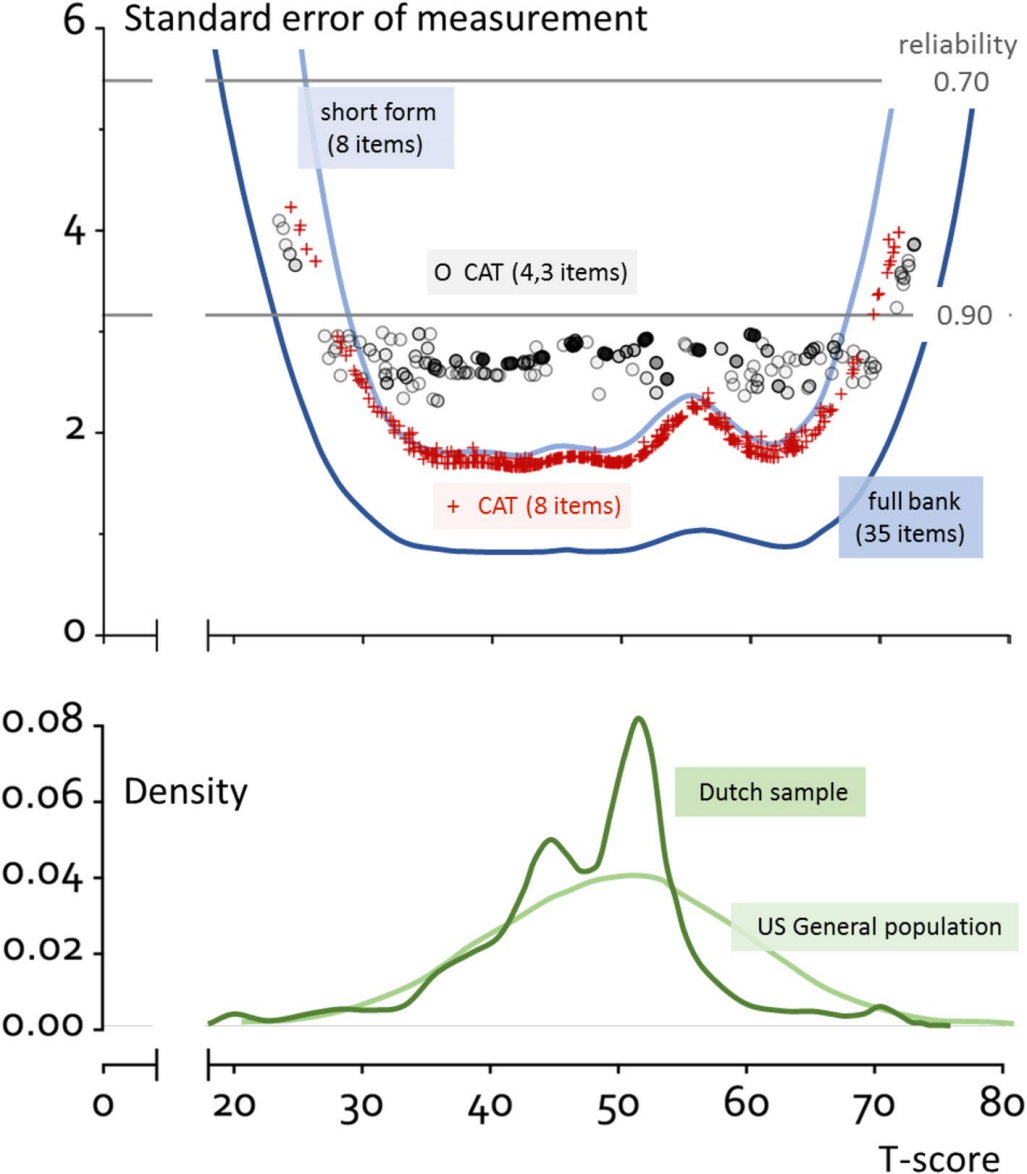
Reliability of the PROMIS V2.0 item bank Satisfaction with Participation in Social Roles and Activities when using different applications (full item bank, short form and CATs (the open circle symbols represent the standard CAT (shading represents many of the same scores) and the plus symbols represent the fixed 8-item CAT)) and distribution of T-scores (based on full item bank) in the population. For *n* = 28 theta could not be estimated and CAT theta scores were set to − 4 (*T*-score 10, *n* = 12) or 4 (*T*-score 90, *n* = 16) in both simulated CATs (these persons are not shown in the plot)

Figure 2 shows the standard error across *T*-scores for the full item bank, the short form, and the two simulated CATs. The full item bank (44 items) had a reliability of > 0.90 for 97% of the participants. The short form (8 items) had a reliability of > 0.90 for 95% of the participants. Using the standard CAT with SE = 3 and max 12 items, a reliability of > 0.90 was obtained for 94% of the participants with an average of 4.3 (range 3–12) items. Using a fixed 8-item CAT, a reliability of > 0.90 was obtained for 93% of the participants.

The mean *T*-score of the study sample (based on the full item bank, obtained from https://www.assessmentcenter.net/ac_scoringservice, using US item parameters) was 47.5 (SD 8.3), range of 20.4–70.8.

## Discussion

We validated the Dutch-Flemish PROMIS item banks V2.0 Ability to Participate in Social Roles and Activities and V2.0 Satisfaction with Social Roles and Activities in a Dutch general population. It comprises (after Spanish) the second foreign language validation and the first validation in the Netherlands. We found sufficient unidimensionality, no local dependence, sufficient monotonicity, good IRT model fit, and a high reliability across a wide range of the construct for both item banks. We found no evidence for DIF due to age, gender, education, region, ethnicity, or language.

For both item banks, we found CFI and TLI values higher than the minimum criteria of 0.95, and comparable to those found in the first validation study, performed in the US and Spanish population [19]. However, the RMSEA was higher than the maximum criterion of < 0.06 (0.11 for both item banks). The RMSEA was not reported for the US and Spanish population. A high RMSEA has been reported for many other PROMIS item banks [56–60]. It has been suggested that traditional cutoffs and standards for CFA fit statistics are not suitable to establish unidimensionality of item banks measuring health concepts [61] and that the RMSEA is sensitive to model complexity (number of estimated parameters) and skewed data distributions [61], the latter being the case in health concepts. Reise et al. have found the RMSEA statistic to be problematic for assessing unidimensionality of health concepts, and considered the SRMR more promising to determine whether a scale is ‘unidimensional enough’ [62].

Four items of the Ability to Participate in Social Roles and Activities item bank and two items of the Satisfaction with Social Roles and Activities item bank showed poor model fit. These items have low to moderate item slopes and low to moderate item information as compared to the other items (data not shown), which implicates that they do not have a high probability to be selected in a CAT. Therefore, these items will likely not cause any problems in CAT administrations. We prefer to keep them in the item bank at the moment because they may have higher information value in specific populations, such as patient groups. This has to be examined in future studies.

We found high reliability of the full item banks, short forms, and simulated CATs. A reliability of > 0.90 (which has been considered a minimum requirement for use of PROMs in individual patients [63]) was found in 85% and 97% of the participants for the 8-item short form, in 82% and 95% of the participants for the 8-item CAT, and in 86% and 94% of the participants for the standard CAT (mean 4.7 and 4.3 items) for the Ability and Satisfaction bank, respectively. These results show two things: First, the short forms and fixed 8-item CATs perform similar in this population. This was to be expected because the short forms include the items that best cover the full construct in the general population. Our results therefore confirm the validity of the short forms. One should keep in mind, however, that in clinical populations, with lower scores on average, short forms may perform less well because their content is then suboptimal. Second, standard CATs perform as well as short forms in this population, but use on average only four to five items instead of eight. In fact, 71% and 54% of the participants needed to complete only two to three items of the Ability and Satisfaction item bank, respectively.

This study has limitations. Although the agreed maximum of 2.5% deviation was met, the study sample was not perfectly representative of the Dutch general population: middle aged people, middle educated people, Western immigrants, females, and participants from the Western part of the Netherlands were slightly overrepresented. This will most likely not have affected the model parameters, since no DIF was found for age, gender, education, region, and ethnicity. The US sample was on average older, contained more women and more highly educated people than the Dutch sample. This will most likely not have affected the DIF for language results, since no DIF was found for age, gender, and education.

With regard to the IRT analyses, it is not clear what the best estimation method is to estimate theta scores. We used maximum likelihood (ML) estimations, while the US PROMIS CAT software uses Expected A Priori (EAP). However, EAP pulls theta estimates towards the center of the population distribution, which may introduce bias in people with extreme responses [55]. For example, we found that respondents with the highest possible scores on almost all items were given a *T*-score of about 68 with EAP, where a *T*-score of 80 or 90 would have been more appropriate. Similar findings were found for the Dutch-Flemish PROMIS V1.0 Anxiety item bank [60]. With ML, however, a theta for participants with response patterns that exclusively comprise extreme responses cannot be estimated at all, which also creates a problem. In this study, we set the possible scale boundaries in the CAT simulation to − 4 to 4, whereby people who score 1 or 5 on all CAT items get a theta score of − 4 or 4, which is equivalent to a *T*-score of 10 and 90, respectively. Another possibility, used for the Dutch-Flemish PROMIS Anxiety item bank [60], is to use another estimation method, such as maximum a posteriori to estimate thetas for participants with extreme responses [47]. More research is recommended to find the optimal approach to estimate theta scores.

An interesting observation in this study was that the average *T*-score of the Satisfaction item bank was 47.5, while an average of 50.0 was to be expected in a general population sample (the average *T*-score of the Ability item bank was 50.6). This may indicate that Dutch people are, on average, less satisfied with their level of participation than US people, but further analyses are needed to explore possible alternative explanations.

In conclusion, the Dutch-Flemish PROMIS item banks Ability to Participate in Social Roles and Activities and Satisfaction with Social Roles and Activities showed sufficient psychometric properties in the general Dutch population. However, test–retest reliability and responsiveness need to be assessed in future studies. These item banks are now ready for use as CAT in research and clinical practice and will be made available through the Dutch-Flemish Assessment Center (http://www.dutchflemishpromis.nl). PROMIS CATs allow reliable and valid measurement of participation in an efficient and user-friendly way with limited administration time.
